# Response to the letter to the editor: Appraisal of suspicious for malignancy cytology: nuclear characteristics deserve special attention in reported cytology analysis - real-world scenario cohort in thyroidology

**DOI:** 10.20945/2359-4292-2026-0003

**Published:** 2026-01-28

**Authors:** Fabiane Carvalho Macedo, Ricardo Luiz Costantin Delfim, Fernanda Vaisman

**Affiliations:** 1 Endocrinologia, Faculdade de Medicina, Universidade Federal do Rio de Janeiro, Rio de Janeiro, Brazil; 2 Instituto Nacional de Câncer, Rio de Janeiro, RJ, Brazil; 3 DASA, Rio de Janeiro, RJ, Brazil; 4 Private Practice, Rio de Janeiro, RJ, Brazil

Dear Editor,

We thank Dr Ilker Sengul and Dr Demet Sengul for their thoughtful and insightful comments ^(1)^ on our recently published manuscript, “In suspicious for malignancy thyroid nodule aspirates, nuclei characteristics deserve special attention in reported cytology analysis - real world scenario cohort” ^(2)^. Our study highlights that nuclear features may refine cytological interpretation within the Suspicious for Malignancy (SFM) Bethesda category, and we welcome the opportunity to address the points raised

Regarding the exceptionally high risk of malignancy (ROM) observed the correspondents correctly noted the remarkably high risk of malignancy (98%) reported in our SFM cohort, exceeding the mean ROM described in the 2023 Bethesda System for Reporting Thyroid Cytopathology (TBSRTC) ^(3)^. As detailed in our Methods and Discussion, this finding reflects the “real-world surgical series” nature of our cohort, in which all SFM nodules were managed surgically and confirmed histologically. This introduces a natural selection bias favoring malignancy confirmation, similar to other surgical cohorts in the literature reporting ROMs between 91% and 100% ^(4)^. Therefore, the elevated ROM observed should not be interpreted as a discrepancy in diagnostic thresholds but as a feature of a highly selected, clinically indicated population.

Qualitative versus quantitative analysis of cytological criteria was another raised issue in the letter. We agree that direct slide reassessment and quantitative morphometric analysis might further clarify the weight of each nuclear parameter. Nevertheless, the qualitative review of finalized cytology reports was a deliberate methodological choice, designed to emulate a genuine clinical setting in which treatment decisions rely on written diagnostic impressions. Despite this constraint, our statistical analysis demonstrated that micronucleoli and oval/irregular nuclei were independently associated with malignancy, increasing its likelihood nearly thirteenfold and fivefold, respectively. These findings underscore the robustness of these parameters, even in report-based analyses, and justify future prospective studies with centralized cytopathologic review.

On possible sources of diagnostic bias, we acknowledge the point regarding the potential influence of the operator’s experience. In our study, the aspirates were obtained by a single radiologist with over 25 years of thyroidology experience, which indeed may have contributed to the low false-positive rate and high ROM observed. However, this consistency enhances the internal validity of the findings by minimizing procedural variability. It is also noteworthy that FNAB material was processed and analyzed by multiple cytopathologists across different laboratories, yet the false-positive rate remained only 2%, comparable to rates reported internationally.

Nuclear features and diagnostic thresholds, were our main finding: the diagnostic strength of micronucleoli and irregular/oval nuclei as discriminators of malignancy within the SFM category. As they correctly observed, traditional features such as nuclear grooves and pseudoinclusions, though highly frequent, lacked discriminatory value in our cohort, as they appeared in both benign and malignant cases. This observation reinforces the notion that not all classical features carry equal predictive power, and supports refining cytological emphasis toward those features that better correlate with true malignancy. Additional multicenter, quantitative, and possibly molecularly integrated studies are warranted to determine how many and which nuclear features should be minimally required to ensure diagnostic precision within the SFM category ^(5)^.

Final considerations: we appreciate the correspondence and the shared interest in refining the cytological evaluation of thyroid nodules. Our study reinforces that even in a qualitative, real-world analysis, micronucleoli and nuclear irregularity emerge as highly specific and reproducible markers of malignancy in the SFM Bethesda category. These findings may serve as a suggestion for cytopathologists, especially in settings where molecular testing is not readily available. We thank the authors for their valuable observations, which further highlight the relevance of continuous refinement in thyroid cytopathology and the importance of maintaining close collaboration between pathologists, radiologists, and clinicians.

## Data Availability

datasets related to this article will be availa-ble upon request to the corresponding author.
